# Words matter, humanity matters: alienating non-citizens from the COVID-19 vaccine

**DOI:** 10.1136/postgradmedj-2021-140212

**Published:** 2021-05-26

**Authors:** Simar Singh Bajaj, Lucy Tu, Fatima Cody Stanford

**Affiliations:** History of Science, Harvard College, Cambridge, Massachusetts, USA; Department of Sociology, Harvard College, Cambridge, Massachusetts, USA; Departments of Medicine and Pediatrics, Massachusetts General Hospital and Harvard Medical School, Boston, Massachusetts, USA

‘Of or belonging to another, not one’s own, foreign, strange.’

From the Latin *alienus*, the etymology of the word ‘alien’ signifies much of what the word connotes: a certain unnatural and inhuman nature. Nonetheless, ever since the Alien and Sedition Acts in 1798, the dehumanising term ‘alien’ has repeatedly been used to refer to immigrants in the USA. On his first day in office, President Biden sent Congress the US Citizenship Act of 2021, which notably sought to change the term ‘alien’ to ‘non-citizen’ in our immigration laws. Much attention, therefore, has been given to this change and its implications within the realm of immigration, but we must also recognise the importance of similar semantic alterations within healthcare. For instance, the Affordable Care Act (ACA) repeatedly refers to ‘non-citizens’ as ‘aliens,’ and such terminology is ubiquitous throughout health policy and the literature more broadly. Eliciting notions of segregation, the term ‘alien’ relegates important communities to a second-class status. The COVID-19 pandemic has exacerbated deep-rooted fissures of trust in the federal government and healthcare institutions, as demonstrated by a palpable hesitancy to receive the three authorised SARS-CoV-2 vaccines among non-citizen communities.^1 2^ In our efforts to curb the COVID-19 pandemic, we cannot permit our diction to further intensify bias and, in turn, alienate immigrants from vaccination.

Already, non-citizens in the USA face difficulties as they endeavour to navigate our complex healthcare system. These realities manifest themselves in disproportionately low levels of health insurance among non-citizens: 77% of lawfully present immigrants and 55% of undocumented immigrants as compared with 91% of citizens.^3^ While undocumented immigrants are entirely ineligible for Medicaid and ACA coverage, lawfully present immigrants are often precluded from these federal programmes because of fear, confusion and literacy challenges, as well as worries about being labelled as a ‘public charge’ (ie, receiving government benefits can make one ineligible for a green card or visa). Unfortunately, the prior administration empowered an Immigration and Customs Enforcement agency that aggressively targeted non-citizens, and, more broadly, our political climate has elevated rhetoric that voraciously maligns all immigrants. As such, it should come to no surprise that immigrants of all documentation statuses have quietly retreated from the public sphere and the healthcare system altogether.^1^ Countless reports have found that non-citizens increasingly avoid scheduling doctor’s appointments and refuse to answer the door for home health visits, which may help to explain why immigrants are less likely to receive preventive care services and are more likely to suffer from chronic diseases.^1 4 5^ While it may be secondary to challenges regarding access, exorbitant costs associated with care, or an unwillingness to put themselves and their families at risk,^4^ the health consequences are disastrous. In the context of COVID-19, non-citizens may avoid seeking medical advice until the last possible moment when the virus has already wrought immense damage on their bodies. Alienated from traditional avenues of care, non-citizens are often caught only in the fraying safety nets of urgent care clinics and emergency rooms with their severely exacerbated conditions.

We have already seen the consequences of such disparities as it relates to the pandemic. Constituting 13.7% of the US population, immigrant essential workers represent 16.3% of essential healthcare operations, 18.4% of essential retail and 20.2% of essential services, disproportionately serving as frontline personnel and sustaining countless industries on the backs of their labour.^6^ Whether it be this work as essential workers or high rates of poverty and other social risk factors, immigrants are at least twice as likely to be infected with COVID-19 as native-born individuals and face significantly higher mortality rates.^1 7^ For instance, in the Dallas Fort-Worth Area, which sees one of the largest populations of undocumented immigrants in the nation, middle-aged Latino men are eight times more likely to die from COVID-19 than their non-Latino white peers.^2^ While immigrants do not necessarily have significantly higher rates of underlying health conditions,^8^ various structural barriers and injustices prevent non-citizens from accessing care, contributing to these higher rates of infection and worse outcomes.

These challenges and the resultant adverse health consequences can erode trust among non-citizens in health systems and federal institutions. Trust is broken in wake of discrimination in clinics. Trust is broken when non-citizens, without insurance, have to pay exorbitant sums to access healthcare. Trust is broken when trips to the hospital put one at risk of being deported. Trust is broken when non-citizens see community members dying needlessly from COVID-19. In a pandemic that has burdened immigrants in particular, subtle mental assaults through stigmatising language only further deteriorate trust. Indeed, the term ‘alien’ implicitly removes non-citizens from the healthcare system and risks excluding them from the COVID-19 vaccination rollout, exacerbating existing structural issues such as limited vaccine availability in these communities.

It is already well known that labelling individuals as ‘illegal aliens’ subjects them to more prejudice and discrimination than does the term ‘non-citizens’.^9^ Indeed, one study found that mental health professionals who thought about Latino immigrants as ‘undocumented immigrants’ viewed them more positively than those asked to think about Latino immigrants as ‘illegal aliens’.^10^ This finding should come to no surprise given that the derogatory term ‘alien’ defines someone *by* their immigration status rather than as a person *with* an immigration status. While ‘non-citizen’ does not entirely resolve the matter of people-first language, it represents a crucial step forward and conveys greater humanity to these individuals. If we cannot purge ‘alien’ from the medical vocabulary entirely, we betray the foundational ideal of equal healthcare for all and turn a blind eye to non-citizens, who represent 14% of the US population.

Certainly, President Biden’s efforts to remove ‘alien’ from our immigration laws is a long-overdue first step to mitigate bias and build trust, but we must broaden our vision towards all realms, including healthcare. The federal government represents the face of the COVID-19 vaccine rollout, yet non-citizens largely do not trust the government to protect them and their communities. This paucity of trust is complex and multifactorial, and revamping diction within complicated pieces of legislation may not have any immediate implications for rebuilding that faith. But the words that pervade policy—and their connotations—set the tone for how we collectively address these communities, as well as the dignity and respect they receive. A semantic transition towards ‘non-citizens’ may ultimately beget public health messaging which comes from bilingual community leaders, assurances that vaccination is free and does not carry a deportation risk, and local efforts to make the vaccine accessible to all immigrants. These steps, in turn, may engender the political will to combat structural barriers that non-citizens face in navigating health institutions. At the end of the day, words matter, humanity matters. During a pandemic indifferent to matters of citizenship, we must make sincere overtures to bridge access to care and deracinate stigmatising, dehumanising language from our vocabulary.
